# Authors’ reply

**DOI:** 10.4103/1817-1737.69122

**Published:** 2010

**Authors:** Ali I Al-Haqwi, Hani Tamim, Ali Asery

**Affiliations:** *Family and Community Medicine, King Saud Bin Abdul Aziz University for Health Sciences, Riyadh, Saudi Arabia. E-mail: alhaqwi@gmail.com*; 1 *Epidemiology and Biostatistics, King Saud Bin Abdul Aziz University for Health Sciences, Riyadh, Saudi Arabia*; 2 *Department of Pediatrics Gastroenterology, College of Medicine, King Fahad Medical City, Riyadh, Saudi Arabia*

Sir,

Many thanks for your comments.[1] I do appreciate your feedback on our work very much. I agree with you that this serious behavior affects the health of our future doctors.[2] Smoking could also point to the need of psychological and counseling support programs.

I support your idea of having a well-established counseling program for medical students to promote their health and to prevent them from being involved in smoking or even more serious behaviors such as substance use.

Best Regards.
